# 5-HT1B receptor agonists promote Schwann cell myelination

**DOI:** 10.1371/journal.pone.0345946

**Published:** 2026-03-27

**Authors:** Yuka Kobayashi-Ujiie, Keiko Hiraki-Kamon, Shuji Wakatsuki, Toshiyuki Araki

**Affiliations:** Department of Peripheral Nervous System Research, National Institute of Neuroscience, National Center of Neurology and Psychiatry, Tokyo, Japan.; NYU Grossman Long Island School of Medicine, UNITED STATES OF AMERICA

## Abstract

Congenital demyelinating peripheral neuropathy causes severe sensorimotor defects, affecting patient mobility. To identify therapeutic compounds for peripheral hypomyelinating neuropathy, we performed an unbiased screening of annotated compound library by in vitro myelination culture model using Trembler (Tr-Ncnp) -derived dorsal root ganglia explants. Among the compounds identified in the screening, we confirmed that zolmitriptan, a 5-hydroxytryptamine 1B/1D receptor agonist, promotes myelination in culture. Zolmitriptan up-regulated myelin-related genes in primary Schwann cells and increased proliferating Schwann cells along axons in DRG explant cultures of Tr-Ncnp mice. Furthermore, we observed that chronic administration of zolmitriptan ameliorates sensorimotor dysfunction in Tr-Ncnp mice. Collectively, these results suggest that 5-HT1B/1D receptor agonists may increase Schwann cells participating in myelination, and consequently ameliorate myelination impairment in peripheral neuropathy.

## Introduction

Myelin is a lipid-rich ionic insulator that wraps around the axon and is essential for rapid saltatory conduction of neuronal electrical signaling [1,2]. In the peripheral nervous system (PNS), myelin is formed by Schwann cells, which are PNS-specific glial cells. Schwann cells are also important for PNS development [3]. Genetic mutation in genes encoding molecules related to Schwann cell structure or metabolism are known to cause peripheral nerve demyelinating disease, including Charcot Marie Tooth disease (CMT), also known as hereditary motor and sensory neuropathies [4]. CMT is the most common hereditary neurologic disorder affecting at least 1 in 2,500 individuals without any disease-specific therapies. Among the diverse genes causing CMT, a major category is CMT1, dominantly inherited demyelinating type of CMT. The majority of CMT1 cases are associated with the peripheral myelin protein 22 (PMP22) gene [5]. PMP22 is a transmembrane glycoprotein, primarily expressed in the compact myelin of the peripheral nervous system [6]. The expression level of PMP22 must be tightly regulated, as both increased and decreased levels of the PMP22 gene product result in dysmyelination [7,8]. Hereditary neuropathy with liability to pressure palsies (HNPP) and Charcot-Marie-Tooth disease type 1E (CMT1E) are both associated with PMP22 mutations but exhibit distinct pathogenic mechanisms [9]. HNPP results from PMP22 haploinsufficiency, typically due to the deletion of one allele, leading to reduced PMP22 expression and subsequent myelin instability. In contrast, CMT1E arises from point mutations in PMP22 that alter protein function rather than simply reducing or increasing its expression. The TremblerJ (TrJ) mouse model carries a specific PMP22 mutation (Leu16Pro) that leads to defective myelination but does not model PMP22 overexpression or underexpression [10]. While PMP22 levels may appear lower in TrJ mice, this likely reflects impaired myelin formation rather than direct downregulation of PMP22 expression. Other models with decreased PMP22 expression include Trembler (Tr-Ncnp) mice, which have an exon 4 deletion in the PMP22 gene [11]. Tr-Ncnp mice also exhibit impaired sensorimotor function due to a significant loss of myelin specific to peripheral nerves, similar to the clinical features observed in CMT1 or HNPP patients. We previously observed that DRG explant culture derived from Tr-Ncnp mice show reduced myelin formation compared to those from wild-type mice [12]. Here, we performed screening of annotated compounds to promote myelination of Tr-Ncnp nerves in culture, and identified zolmitriptan, a known serotonin (5-hydroxytryptamine, 5-HT1B/1D) receptor agonist, increased the number of myelin segments. We found that 5-HT1B/1D receptor agonists promoted myelination in DRG explant culture from wild-type mice in a dose-dependent manner. The 5-HT1B/1D receptor is known to be expressed in Schwann cells and DRG neurons. We also found that zolmitriptan up-regulated myelin-related genes in primary Schwann cells, and increased proliferating Schwann cells along axons in DRG explant cultures of Tr-Ncnp mice. Furthermore, we observed that chronic administration of zolmitriptan ameliorates sensorimotor dysfunction in Tr-Ncnp mice. These results collectively suggest that 5-HT1B/1D receptor agonists may increase the number of Schwann cells participating in myelination, and consequently ameliorate myelination impairment in peripheral neuropathy.

## Materials and methods

### Animals

All the experimental procedures using mice and rats were approved by the Committee on Ethical Issues in Animal Experiments at National Center of Neurology and Psychiatry (approval number: 2023007). Mice and rats were purchased from CLEA Japan. All experimental animals were kept at room temperature (23–24°C, 60–70% humidity), on a 12-hour light/dark cycle, with free access to water and food. All mice used in experiments were on C57BL/6 background. To obtain heterozygous Tr-Ncnp mice, homozygous female mice were crossed with wild-type male mice, or embryos were generated by in vitro fertilization using sperm derived from homozygous males and eggs from wild-type (C57BL6) females and then transferred to pseudo-pregnant mothers. Generated Tr-Ncnp mice were genotyped by PCR.

For harvesting tissues for primary cultures, mice were euthanized by cervical dislocation. For histological analysis, mice were euthanized by medetomidine-midazolam-butorphanol overdosage.

### Compound administration

Zolmitriptan (Tokyo Chemical Industry, Z0024), sumatriptan succinate (Tokyo Chemical Industry, S0851), SB224289 (Tocris, 1221), candesartan (Tokyo Chemical Industry, C2635), telotristat (Sigma-Aldrich, 1033805-28-5), naphazoline hydrochlolide (Tokyo Chemical Industry, N0542), 3-hydroxytyramine hydrochloride (Tokyo Chemical Industry, A0305), pancuronium dibromide (Abcam, ab120535) and phenytoin sodium (Tokyo Chemical Industry, D1331) were dissolved in DMSO and then diluted in sterile PBS for cell culture applications or saline for in vivo mouse administration. For administration to mice, zolmitriptan was intraperitoneally injected at 30 mg/kg according to previous reports [13]. We did not observe abnormal behaviors elicited by zolmitriptan administration. For examining effect on mouse behavior, zolmitriptan was administered three times a week for four weeks prior to the examinations.

### In vitro myelination from mouse DRG explant culture

DRG explants were cultured as previously described [12,14–16]. Briefly, DRGs were dissected from E12 mouse embryos, cultured on 24 well plates coated with poly-L lysine (100 μg/ml, Sigma Aldrich) and laminin (Cultrex mouse laminin I, 2.5 μg/ml, R&D systems, #: 3400-010-01). These cultured DRGs were maintained in MACS Neuro Medium supplemented with 1% MACS NeuroBrew-B21 (Miltenyi Biotec, 130-093-566), 1:200 Glutamax, 50 U/ml Penicillin/Streptomycin and 100 ng/ml 2.5S NGF. At 5 DIV, the medium was changed to DMEM/F12 (Wako, 048–29785) supplemented with 1:100 N2 supplement (R&D systems, AR009), 50 U/ml Penicillin/Streptomycin and 100 ng/ml 2.5S NGF. At 10 DIV, DRGs were cultured in medium for myelination: DMEM (Sigma, D5030) supplemented with 10% FBS, 1:200 Glutamax, 50U/ml Penicillin/Streptomycin and 100 ng/ml 2.5S NGF, and 50 μg/ml of L-ascorbic acid (Sigma Aldrich).

### Immunostaining (immunocytochemistry and immunohistochemistry)

Immunostaining was performed according to our previous report [12,15]. In DRG explant cultures, myelination was assessed by counting the number of MBP-positive segments within a defined area containing comparable densities of NFM-positive axons. In sciatic nerve sections, myelination was evaluated by counting the number of MBP-positive axons within a defined area containing a comparable number of DAPI-positive nuclei.

### Electron microscopy

To analyze myelin ultrastructure, transmission electron microscopy was performed as described previously [12,15]. Briefly, sciatic nerves were fixed overnight in 1.4% paraformaldehyde (Wako) and 1% glutaraldehyde in PBS, followed by post-fixation with 1% osmium (VIII) oxide solution (FUJIFILM). Samples were then serially dehydrated and embedded in Epon 812 (TAAB). Ultrathin sections were cut and stained with uranyl acetate and lead citrate. Electron micrographs were obtained using a transmission electron microscope (Tecnai Spirit, FEI/Thermo Fisher Scientific). Axonal area and myelinated fiber area were measured for 100 axons using ImageJ software (NIH). Diameters were calculated as equivalent diameters using the formula: 2 × √(area/π). The g-ratio was calculated as the ratio of the inner axonal diameter to the total fiber diameter. Analysis of covariance (ANCOVA) was used to evaluate group differences in the relationship between g-ratio and axon diameter, with axon diameter included as a covariate and treatment group as a fixed factor. An interaction term between axon diameter and group was incorporated to test for differences in regression slopes.

### Edu proliferation assay

Edu incorporation assays were carried out using the Clik-iT EdU labeling Kit (Invitrogen) according to the manufacturer’s instructions. DRGs from Tr-Ncnp mice were cultured in myelination medium. After 6 days, Edu was added at a final concentration of 50 µM to the culture 2 hours before PFA fixation for detection. The number of Edu-positive cells was counted under a microscope in eight randomly selected fields per well for each condition, which was then divided by the total number of Hoechst-positive cells to obtain the percentage of Edu-positive cells.

### Schwann cells culture

To perform primary rat Schwann cell culture as described in previous reports [14–16], sciatic nerves were isolated from neonatal rats at postnatal day 2. The sciatic nerves were dissociated with collagenase (Worthington Biomedical, CLS-1,) and dispase (Collaborative, 40235). To remove fibroblasts, the culture was treated with 10 μM cytosine arabinoside (Wako), followed by complement-mediated cytolysis using anti-Thy1.1 (Serotec, Oxford, UK) and rabbit complement (Cappel laboratories, Cochranville, PA, USA). For expansion, Schwann cells were cultured in DMEM (Wako, 044–29765) supplemented with 10% FBS, 2 μM forskolin (Sigma Aldrich, F6886), and 10 ng/ml Hereglin β1 (EGF domain) (Sigma, H7660), and then used for experiments after at least 3 passages.

Mouse primary Schwann cells from genotyped Tr-Ncnp or wild-type mice were prepared from DRG explant cultures. All DRGs isolated from a single mouse embryo (E12) were explanted in one well of a 6 well plate coated with poly-L lysine (100 μg/ml, Sigma Aldrich) and laminin (Cultrex mouse laminin I, 2.5 μg/ml, R&D systems, #: 3400-010-01). These DRGs were cultured in the medium as described above. At 8 DIV, cells were detached using trypsin-EDTA (Wako, 209–16941), and passaged onto non-coated plates. They were then cultured with DMEM (Wako, 044–29765) supplemented with 10% FBS, 2 μM forskolin, and 10 ng/ml Heregulin β1.

### Immunoblotting

Total protein was extracted by SDS-containing sample buffer (2% SDS, 10% Glycerol, 50 mM Tris-HCl, PH = 6.8) with protease and phosphatase inhibitor cocktail (Nacalai Tesque). Lysate containing 10 μg proteins per sample was run by 10% SDS-PAGE and followed by immunoblotting. Immunoreactivity was visualized using a horseradish peroxidase (HRP)-conjugated secondary antibody and a chemiluminescent substrate (FUJIFILM, ImmunoStar). Chemiluminescent images were captured by FUSION SOLO S (VILBER). Quantification of band intensity in captured images was analyzed with Image J software (National Institutes of Health, Bethesda, MD). β-actin was used as a loading control.

### Quantitative real-time PCR

Cultured cells and mouse sciatic nerve were collected. Total RNA was purified from each sample using TRI Reagent (Molecular Research Center). 1 μg of total RNA was reverse transcribed by ReverTra Ace (TOYOBO) using random primers. qPCR reactions were performed with the THUNDERBIRD qPCR Mix (TOYOBO) using the Applied Biosystems Prism model 7300 sequence detection instrument according to a standard SYBR green detection protocol. The sequences used for PCR amplification are as follows:

Ms_β-actin forward 5’-GGCTGTATTCCCCTCCATCG, reverse 3’-CCAGTTGGTAACAATGCCATGT-5’
Ms_5-HT1BR forward 5’-CGCCGACGGCTACATTTAC −3’, reverse 5’-TAGCTTCCGGGTCCGATACA −3’
Rat_β-actin forward 5’-AGGCCATGTACGTAGCCATCCA-3’, reverse 5’-TCTCCGGAGTCCATCACAATG-3’
Rat_5-HT1BR forward 5’-ATGGAGGAGCAGGGTATTCAG-3’, reverse 5’-CGCTAACAAAGCAACCAGCA-3’
Rat_Oct6 forward 5’-TACCGCGAAGTGCAGAAGC-3’, reverse 5’- CGTGGGTAGCCATTGAGGG-3’
Rat_p75NTR forward 5′-CCCTCAAGGGTGATGGCAACCTCT-3′, reverse 5′-TGTCAGCTCTCTGGATGCGTCGC-3′

### Antibodies

The antibodies used in each experiment were as follows: anti-5-HT1B receptor (WB, rabbit polyclonal antibody, Proteintech, 22189–1-AP), anti-Myelin basic protein (ICC and IHC, rat monoclonal antibody, Merck, MAB386); anti-Neurofilament M (ICC and IHC, rabbit polyclonal antibody, Merk, AB1987); anti-β-actin (WB, rabbit polyclonal antibody, BioLegend, 622101, RRID: AB_315945). For detection, appropriate secondary antibodies coupled to Horseradish peroxidase (HRP) from Jackson Immunoresearch and Alexa fluorophores from Thermo Scientific were used.

### Beam walking

Beam-walking test was performed based on previous reports with slight modifications [17]. The beam consisted of a single wooden long strip (90 cm) with round (30 mm diameter) cross-section, raised 45 cm above the bench surface. The number of slips and the latency to fall from the narrow beam (60 sec as cut-off time) were measured. The beam diameter was set based on behavioral experiments with Tr-Ncnp mice, in which the latency to fall from the beam did not exceed the cut-off time and was not zero seconds. Mice were trained to cross a beam three times on three consecutive days. After training, the administration of zolmitriptan was started. Each mouse was tested three times and the average value was calculated. The effects of the drugs on peripheral nerves were compared before and after four weeks of drug administration. All behavioral examinations were conducted under blinded conditions by experimenters who were unaware of the treatment groups. Tr-Ncnp mice treated with vehicle (saline containing DMSO) served as controls.

### Statistical analysis

All data obtained from each experiment was used. Statistical analyses were performed using GraphPad Prism (GraphPad Software, Inc.). We performed randomization methods in all experiments. Data are presented as mean ± S.D. in all the figures. For comparison between the two groups, the results were analyzed using an unpaired Student’s t-test (two-tailed). For comparison of more than three groups, the results were analyzed using a one-way analysis of variance, followed by post hoc Tukey’s test. P-Values were considered statistically significant at p < 0.05.

## Results

### 5-HT1B/D receptor agonist facilitates peripheral myelination *in vitro*

To identify compounds effective for ameliorating peripheral nerve demyelinating diseases, we applied each of ~400 commercially available annotated compounds to myelination cultures of DRG explants from Tr-Ncnp mice. Each compound was tested in duplicate at single dose (10 μM). DRG explant cultures of Tr-Ncnp mice show only a few short myelin segments with faint myelin basic protein (MBP) immunoreactivity, as described previously [12]. Compounds causing statistically significant increase in the number of MBP-positive segments were regarded positive for the screening. We identified 7 compounds (Table 1) that showed an increase in the number of myelin segments compared to vehicle. Among them, zolmitriptan, phenytoin sodium, and pancuronium dibromide were confirmed in follow-up validation experiments to increase myelin segments. Phenytoin sodium, an antiepileptic drug, exhibits nonlinear pharmacokinetics, which requires blood concentration monitoring in clinical practice during long-term administration [18]. Moreover, long-term treatment has been associated with cerebellar atrophy [19]; therefore, in disease models such as Tr-Ncnp with pronounced tremor, it was judged that accurately assessing drug effects using the current behavioral methods would be difficult. Pancuronium dibromide, which has muscle relaxant effects [20], was administered intraperitoneally at 0.1 mg/kg to Tr-Ncnp mice but resulted in death, and thus further analyses could not be performed.

**Table 1:** List of compounds identified in the primary screening.

compound name	compound function
candesartan	angiotensin II receptor antagonist
zolmitriptan	5-HT1B/1D receptor agonist
telotristat	tryptophan hydroxylase inhibitor
naphazoline hydrochloride	adrenaline receptor agonist
3-hydroxytryramine hydrochloride	dopamine receptor agonist
pancuronium dibromide	acetylcholine receptor antagonist
phenytoin sodium	sodium channel blocker

**Fig 1:**
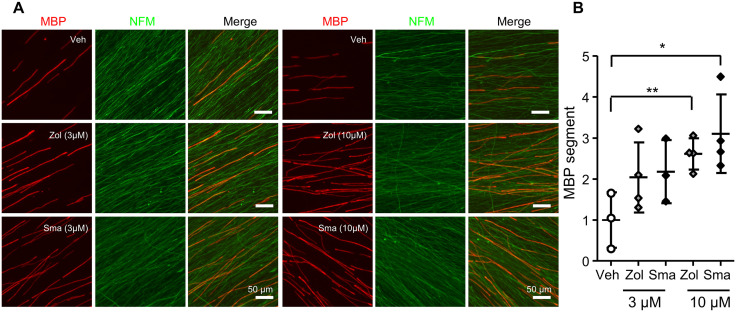
Promoting effects of 5-HT1B receptor agonists on peripheral myelination in a dose-dependent manner. DRG explant cultures from C57BL/6 mice were treated with vehicle or 5-HT1B receptor agonists (zolmitriptan or sumatriptan at 3 μM, 10 μM) for 2 weeks from the start of myelination. **(A)** Representative micrographs of immunocytochemistry analysis using antibodies for myelin basic protein (MBP) and neurofilament M (NFM). Red, MBP. Green, NFM. Scale bar, 50 μm. **(B)** Quantification of the number of MBP segments in each condition. Graphs show values relative to vehicle (myelination media containing DMSO 0.03%), with mean mean± SD. Myelination segments were counted in four randomly selected fields of three to four independent culture experiments performed for each condition. (n = 3-4, **P<0.01, *P <0.05 by unpaired Student’s t-test).

To confirm the effect of zolmitriptan, an agonist for serotonin 5-HT1B/D receptors, on peripheral myelination, we performed DRG explant culture from C57BL6 mice (wild-type). Zolmitriptan dose-dependently increased the number of myelin segments, promoting normal peripheral myelination. Sumatriptan, another agonist for 5-HT1B/D receptor, also induced an increase of myelin segments in a dose-dependent manner (Fig 1A and B). These results suggest that peripheral myelination may be promoted through the 5-HT1B receptor.

### Zolmitriptan ameliorates the tremor phenotype in Tr-Ncnp mice by promoting myelination

**Fig 2:**
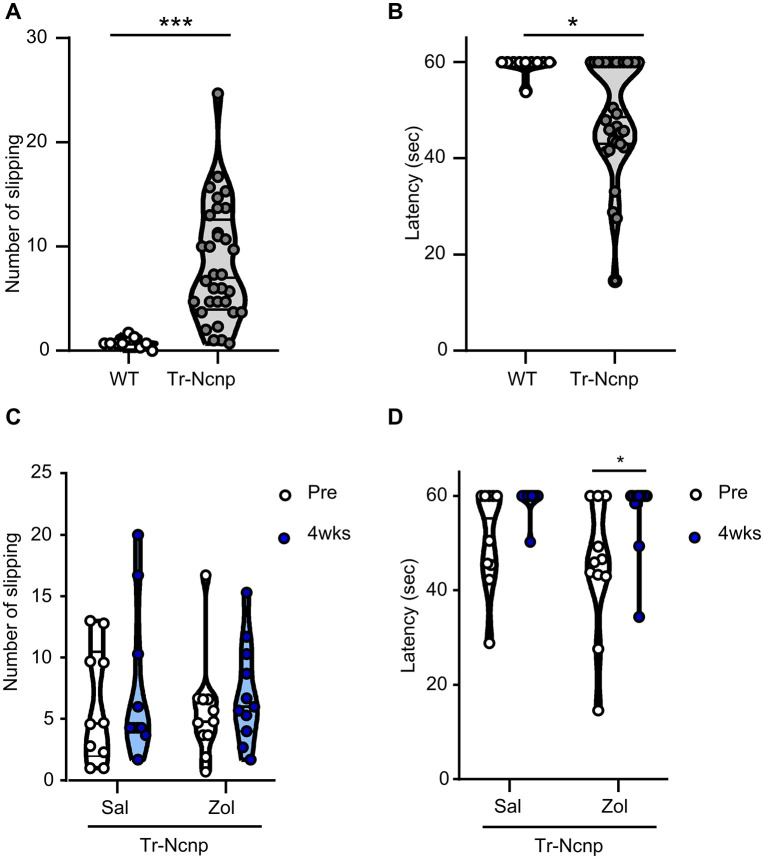
The effect of zolmitriptan on the tremor phenotype in Tr-Ncnp mice. Peripheral nerve function was evaluated by the beam walking test. The number of slips and the time to fall off the narrow beam (latency) were counted for 60 seconds. Each plot represents the average of three trials per mouse. Some data points overlap because the values were identical. **(A, B)** The frequency and latency in 10-week-old C57BL6 (wild-type) and same-aged Tr-Ncnp mice were compared. (n = 10-32, ***P<0.001 and *P<0.05 by unpaired Student’s t-test, mean ± SD). **(C, D)** The effect of zolmitriptan on impaired peripheral nerve function in Tr-Ncnp was assessed by the beam walking test. Zolmitriptan (30 mg/kg, i.p., Zol) or saline (Sal) was administered three times a week for four weeks. (n = 10-11, *P<0.05 by unpaired Student’s t-test, mean ± SD).

We found here that 5-HT1B receptor agonists promote Schwann cell myelination in culture. To examine whether 5-HT1B receptor agonists can ameliorate the disease phenotype of Tr-Ncnp mice in vivo as well, we administered zolmitriptan to Tr-Ncnp mice and analyzed their behavior. We evaluated the behavior of Tr-Ncnp mice with the beam-walking test. Tr-Ncnp mice exhibited an increased number of slips and decreased latency to falling off the narrow beam when compared to wild-type mice (Fig 2A and B). Chronic administration of zolmitriptan (30 mg/kg, i.p., three times a week for four weeks) to Tr-Ncnp mice did not result in significantly improved slip frequency, but these mice showed a longer latency to fall from the narrow beam than prior to treatment (indicated as Pre) (Fig 2C and D). These results suggest that zolmitriptan may be effective in improving the impairment of peripheral nerve function due to PMP22 deletion.

**Fig 3:**
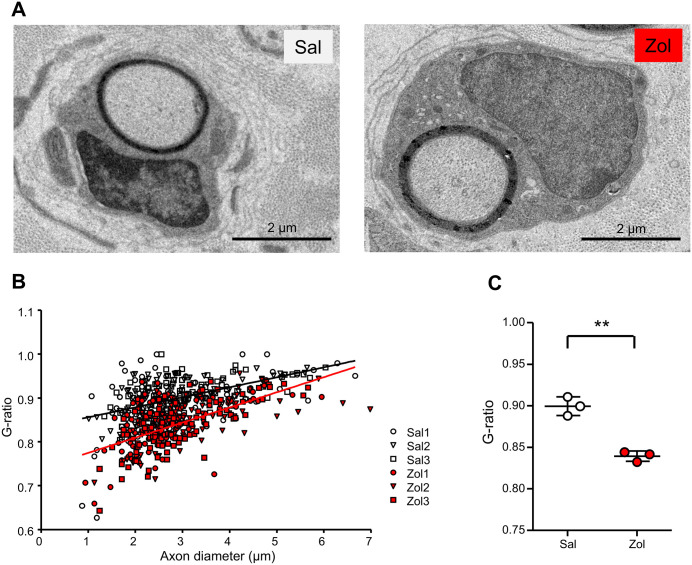
Electron microscopic assessment of zolmitriptan-induced increase of myelination in Tr-Ncnp mice. **(A)** Representative electron micrographs of the sciatic nerve in Tr-Ncnp mice following treatment with zolmitriptan or saline. Zolmitriptan (30 mg/kg, i.p., Zol) or saline (Sal) was administered three times a week for four weeks. **(B)** Scatter plot showing the relationship between axon diameter and g-ratio. The g-ratio was defined as the ratio of the inner axonal diameter to the total fiber diameter. Individual data point represents single axon measured from electron micrograph. Solid lines in the scatter plot indicate linear regression fits for each group. White and red symbols indicate Sal-treated and Zol-treated Tr-Ncnp mice, respectively. **(C)** Graph show the average of g-ratio for each group. (n = 3, **P <0.01 by unpaired Student’s t-test, mean ± SD).

To analyze the mechanism of zolmitriptan-induced amelioration of the Tr-Ncnp mouse behavior phenotype, we analyzed histological changes in peripheral axons by zolmitriptan administration. In immunohistochemical analysis, zolmitriptan-treated mice exhibited a tendency toward increased axonal myelination compared with vehicle-treated controls, although the difference was not statistically significant (S1 Fig). Given this trend toward increased myelination by immunostaining, we next examined myelin morphology in greater detail using electron microscopy (Fig 3A). For each axon, axon diameter was plotted against the corresponding myelin thickness to assess the relationship (Fig 3B). The scatter plot indicates that axons with smaller diameters have lower g-ratios, and both zolmitriptan-treated and vehicle-treated groups exhibit regression lines with positive slopes, indicating that smaller axons tend to be more heavily myelinated. Notably, g-ratio values were shifted downward in zolmitriptan-treated Tr-Ncnp mice compared with controls, with the most pronounced reduction observed in axons of small and intermediate diameters, suggesting enhanced myelination particularly in axons of approximately 2 µm in diameter. Additionally, administration of zolmitriptan significantly reduced average of g-ratio (Fig 3C). These results suggested that zolmitriptan promotes the proper myelin formation around axons.

### 5-HT1B receptor localizes on Schwann cells and neurons in the peripheral nervous system

**Fig 4:**
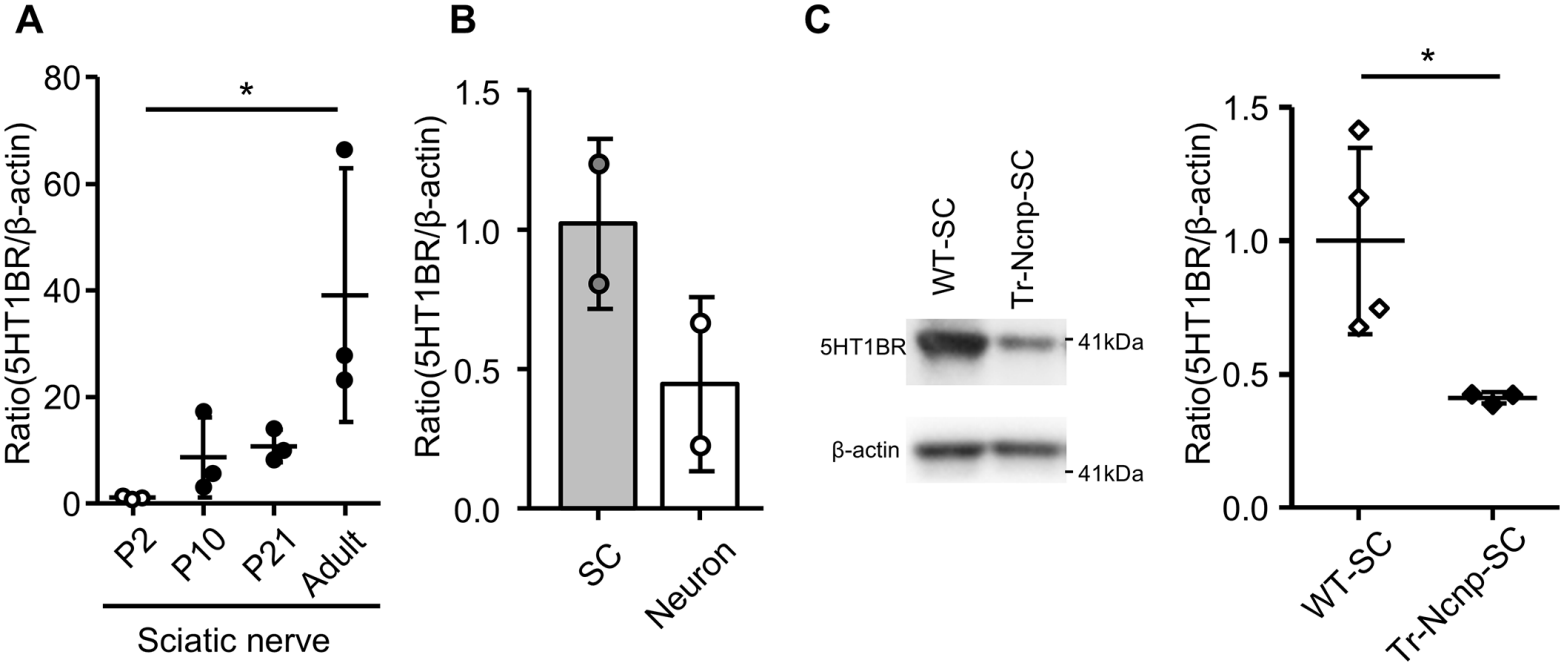
The expression of the 5-HT1B receptor in Schwann cells and neurons. **(A)** The mRNA expression levels of the 5-HT1B receptor relative to β-actin in the sciatic nerve during development were evaluated by quantitative RT-PCR. Individual points indicate the values relative to expression level 2 days after birth. (n = 3, *P <0.05 by one-way ANOVA with Tukey analysis, mean ± SD). **(B)** The mRNA expression levels of the 5-HT1B receptor in primary cultured rat Schwann cells (SC) and in mouse DRG explant culture that were treated with anti-mitotic agents to eliminate proliferative cells (Neuron). **(C)** Protein levels of the 5-HT1B receptor in Schwann cells derived from mouse sciatic nerves were measured by western blot. Quantification of 5-HT1B receptor protein expression levels was normalized to those of β-actin. Each bar shows the mean of relative value compared to C57BL/6 mice (WT) (n = 3-4, *P<0.05 by unpaired Student’s t-test, mean ± SD).

To analyze the mechanism of zolmitriptan-induced amelioration of the Tr-Ncnp mouse behavior phenotype, we performed 5-HT1B receptor expression analysis by employing a culture system. Previous reports have shown that the 5-HT2A receptor is distributed in the sciatic nerve and Schwann cells [21,22], but the localization of other 5-HT receptors in the peripheral nerve remains unclear. We investigated the expression of the 5-HT1B receptor, which is an action point for zolmitriptan, in the peripheral nervous system. By quantitative RT-PCR, 5-HT1B receptor mRNA expression was detected in mouse sciatic nerves and gradually increased with development (Fig 4A). 5-HT1B receptor mRNA expression was also observed in primary rat Schwann cells and mouse DRG neurons (Fig 4B). We also confirmed the expression of 5-HT1B receptor in mouse Schwann cells purified from DRG explant culture. 5-HT1B receptor expression was detected in Schwann cells from both wild-type and Tr-Ncnp mice, but expression was lower in Tr-Ncnp mice when compared to wild-type (Fig 4C, S2 Fig). These results suggest that 5-HT1B receptor agonists promote myelination via the receptor expressed in Schwann cells and peripheral neurons.

### Zolmitriptan increases the number of Schwann cells participating in myelination and promotes Schwann cell differentiation

**Fig5:**
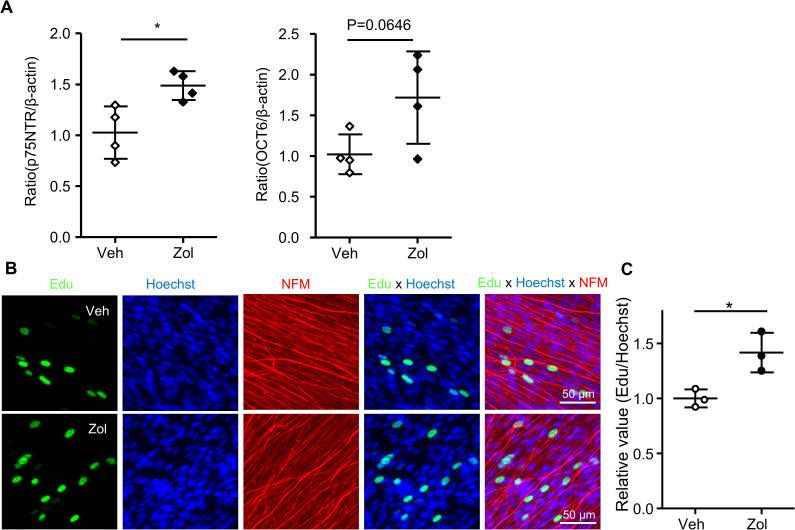
Zolmitriptan promotes Schwann cell differentiation and induces proliferation along axons. **(A)** The mRNA expression of p75NTR and oct6 in primary rat Schwann cell cultures were evaluated by quantitative RT-PCR. Schwann cells were treated with zolmitriptan (10 μM) or vehicle (media containing DMSO 0.01%) (n = 4, *P<0.05 by unpaired Student’s t-test, mean ± SD). **(B)** Edu-positive proliferating Schwann cells were detected by immunocytochemistry in DRG explant culture derived from Tr-Ncnp mice. DRG explant cultures were treated with zolmitriptan (10 μM) or vehicle (media containing DMSO 0.01%) for six days after initiation of myelination. Representative micrographs of proliferating Edu-positive Schwann cells in each treatment are shown. Green, nuclei of Schwann cells which incorporate EdU into DNA; blue, Hoechst-positive Schwann cell nuclei; Red, neurofilament M (NFM); scale bar, 50 µm. **(C)** Quantification of the ratio of Edu-positive Schwann cells in the total number of Hoechst-positive Schwann cells. Edu-positive nuclei were quantified in eight randomly selected fields for each condition. Each bar is the average of three independent experiments and shows relative value compared to the vehicle treatment (n = 3, *P<0.05 by unpaired Student’s t-test, mean ± SD).

To investigate the mechanism underlying facilitation in peripheral myelination by 5-HT1B receptor agonists, we first examined whether the 5-HT1B receptor agonists directly promote the myelinating phenotype of Schwann cells. For this purpose, we applied zolmitriptan to primary Schwann cell culture. We found that zolmitriptan does not affect the expression level of krox20, a transcription factor regarded as the master regulator of Schwann cell myelination. On the other hand, we found that expressions of p75NTR, as well as oct6, which characterize immature Schwann cells, are increased by zolmitriptan (Fig 5A). These results suggest that 5-HT1B receptor agonists may promote myelination by acting on immature Schwann cells prior to myelination.

**Fig 6:**
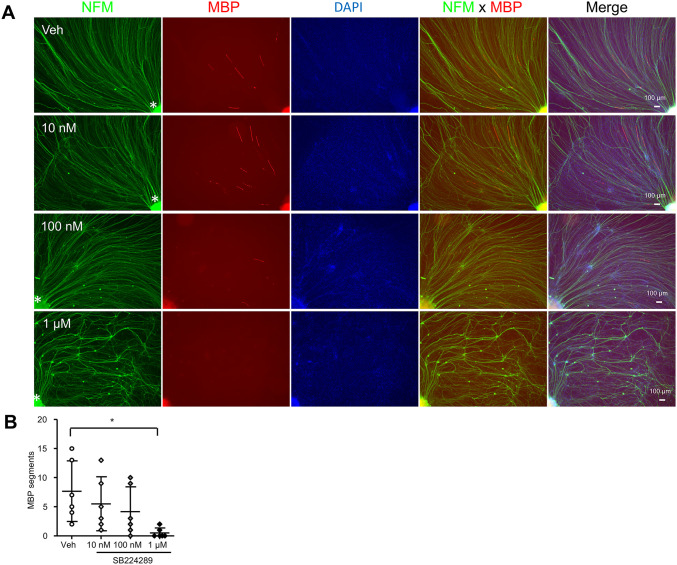
SB224289 treatment decreases the number of Schwann cells in myelination culture. DRG explant cultures from C57/BL6 mice were treated with 5-HT1B receptor antagonists (SB224289 at 10 nM, 100 nM, 1 μM) for 2 weeks from the start of myelination. **(A)** Representative micrographs of immunocytochemistry analysis using antibodies for myelin basic protein (MBP) and neurofilament M (NFM). Red, MBP. Green, NFM. Blue, DAPI. Scale bar, 100 μm. * shows the position of the cell bodies. **(B)** Quantification of the number of MBP segments in each condition. Myelination segments were counted within a constant microscopic field in six independent culture experiments performed for each condition. (n = 6, **P<0.01 by One-way ANOVA, mean± SD).

To gain insights into the mechanism of 5-HT1B receptor-dependent promotion of myelination, we examined the effect of 5-HT1B receptor agonists on Schwann cell proliferation. For this purpose, DRG explant cultures from Tr-Ncnp mice were treated with zolmitriptan for one week followed by Edu labeling. We found that the number of Edu-positive Schwann cells contacting neurites was increased by zolmitriptan (Fig 5B and C). To confirm 5-HT1B receptor-mediated Schwann cell proliferation signaling, we applied a selective 5-HT1B receptor antagonist SB224289 to DRG explants derived from wild-type mice. We found that SB224289 treatment reduces the number of Schwann cells in culture and resultant decrease in axonal density compared to untreated controls. These findings suggest that inhibiting 5-HT1B receptor signaling reduces Schwann cell proliferation or survival, thereby affecting myelination (Fig 6). Collectively, our data suggest that 5-HT1B receptor-mediated signaling may increase the number of Schwann cells participating in myelination, thereby promoting Schwann cell myelination.

## Discussion

In this study, we carried out a screening to identify effective treatments for myelin impairment caused by peripheral neuropathies. To this end, we performed in vitro myelination using DRG explant culture from Tr-Ncnp mice. Our screening results revealed that zolmitriptan, a selective agonist for 5-HT1B/D receptors, significantly increases myelin segments. In addition, zolmitriptan administration ameliorates peripheral nerve dysfunction of Tr-Ncnp mice by promoting myelination. With regard to the mechanism of action, we found that zolmitriptan promotes proliferation of Schwann cells in contact with axons. In the present study, proliferative Schwann cells were quantified on day 6 after the onset of myelination induction, which we consider to correspond to the critical transition phase from proliferative immature Schwann cells to myelinating Schwann cells. At this stage, we observed that the number of Edu-positive Schwann cells closely associated with axons increases following zolmitriptan treatment, suggesting that zolmitriptan enhances the pool of Schwann cells competent to participate in myelination. This finding correlates with the observed upregulation of oct6 and p75NTR mRNA expression in Schwann cell monocultures treated with zolmitriptan.

In the present study, we provide the first evidence that stimulation of Schwann cells with a specific ligand of the 5-HT receptor promotes peripheral nerve myelination. Our current results may suggest a novel avenue for pharmacological intervention to treat peripheral nerve demyelinating neuropathy. Future studies are needed to clarify the physiological and pathological contexts in which this signaling pathway exerts its functions, to define its biological significance. In our beam walking test, the latency to fall of Tr-Ncnp mice was improved by zolmitriptan administration, while the number of slips was not significantly affected. These results may be associated with the partial recovery from peripheral nerve demyelination induced by zolmitriptan administration observed in our histological analysis. Optimizing dosing regimens and administration schedules of zolmitriptan, and exploring potential combinatorial approaches, will be important for maximizing its therapeutic potential as a strategy for peripheral demyelinating diseases.

Previous reports have shown that neuronal activity can affect Schwann cell myelination status [23,24]. Schwann cells express a variety of neurotransmitter receptors, including acetylcholine and glutamate receptors [25,26]. Subcellular signaling elicited by these receptors affects Schwann cell proliferation and differentiation. For example, we previously demonstrated that metabotropic glutamate receptor signaling in Schwann cells regulates their proliferation [16]. Here we showed that zolmitriptan treatment induces expression of p75NTR and oct6. Expression of p75NTR is known to be induced in Schwann cells during early development. P75NTR mediates signaling associated with Schwann cell survival and differentiation [27]. P75NTR null mutant mice show decreased expression of myelin proteins, including MBP and MPZ, and resultant inhibition of remyelination after injury in peripheral nerves [28]. Zolmitriptan-induced p75NTR induction may promote survival and increase the number of Schwann cell that participate in myelination. Oct6, on the other hand, is an important regulator of Schwann cell development [29]. In particular, Oct6 is known to bind to the cis-regulatory element of Krox20 gene, a master regulator of peripheral nerve myelination, and thereby activate its transcription and promote myelination [30]. Zolmitriptan supposedly promotes krox20 transcription via induction of oct6 expression. Previous reports showed that an increase in subcellular calcium ion (Ca^2+^) concentration in Schwann cells may enhance their proliferation and induce expression of krox20 and myelin basic protein to promote differentiation [31,32]. Since subcellular signaling elicited by 5-HT1B receptor activation includes modification of Ca^2+^ levels [33], it is possible that the effect of 5-HT1B receptor agonists on Schwann cell myelination may also be mediated by subcellular Ca^2+^ regulation. Further investigation will be required to clarify the detailed subcellular signaling mechanism elicited by 5-HT receptors in Schwann cells.

## Supporting information

S1 Fig Myelinated axons of sciatic nerves from Tr-Ncnp mice tend to increase by zolmitriptan administration. (A) Representative micrographs of immunocytochemistry analysis using antibodies for myelin basic protein (MBP) and neurofilament M (NFM) in sciatic nerves of Tr-Ncnp mice administered with vehicle or zolmitriptan. Green, MBP. Red, NFM. Blue, DAPI. Scale bar, 50 μm. (B) Quantification of the MBP-positive axon (NFM immunoreactive structure surrounded by MBP immunoreactivity; shown as white arrow head) in shown in A. Individual points show the percentage of MBP-positive axon in a constant area (75 μm x 75 μm). (n = 4, unpaired Student’s t-test, mean± SD).
(TIF)

S2 Fig Uncropped immunoblot images shown in Fig 4C. (TIF)
