# Supplementary figures and images for: 5-HT1B receptor agonists promote Schwann cell myelination
Source: PLoS One. 2026 Mar 27;21(3):e0345946. doi: 10.1371/journal.pone.0345946 (PMC13028473; doi:10.1371/journal.pone.0345946)

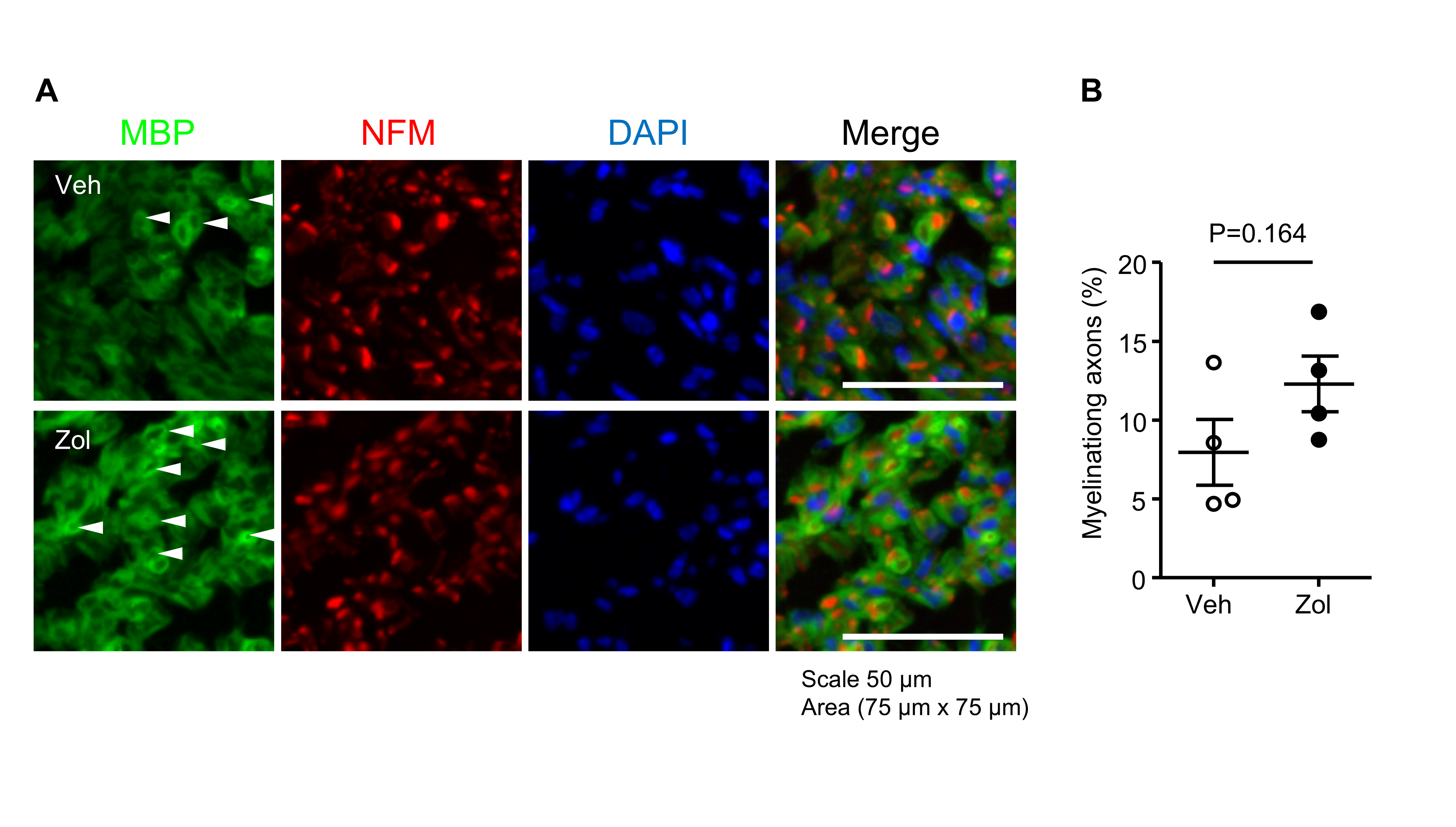

Supplement: S1 Fig — (A) Representative micrographs of immunocytochemistry analysis using antibodies for myelin basic protein (MBP) and neurofilament M (NFM) in sciatic nerves of Tr-Ncnp mice administered with vehicle or zolmitriptan. Green, MBP. Red, NFM. Blue, DAPI. Scale bar, 50 μm. (B) Quantification of the MBP-positive axon (NFM immunoreactive structure surrounded by MBP immunoreactivity; shown as white arrow head) in shown in A. Individual points show the percentage of MBP-positive axon in a constant area (75 μm x 75 μm). (n = 4, unpaired Student’s t-test, mean± SD). (TIF) [file pone.0345946.s001.tif]

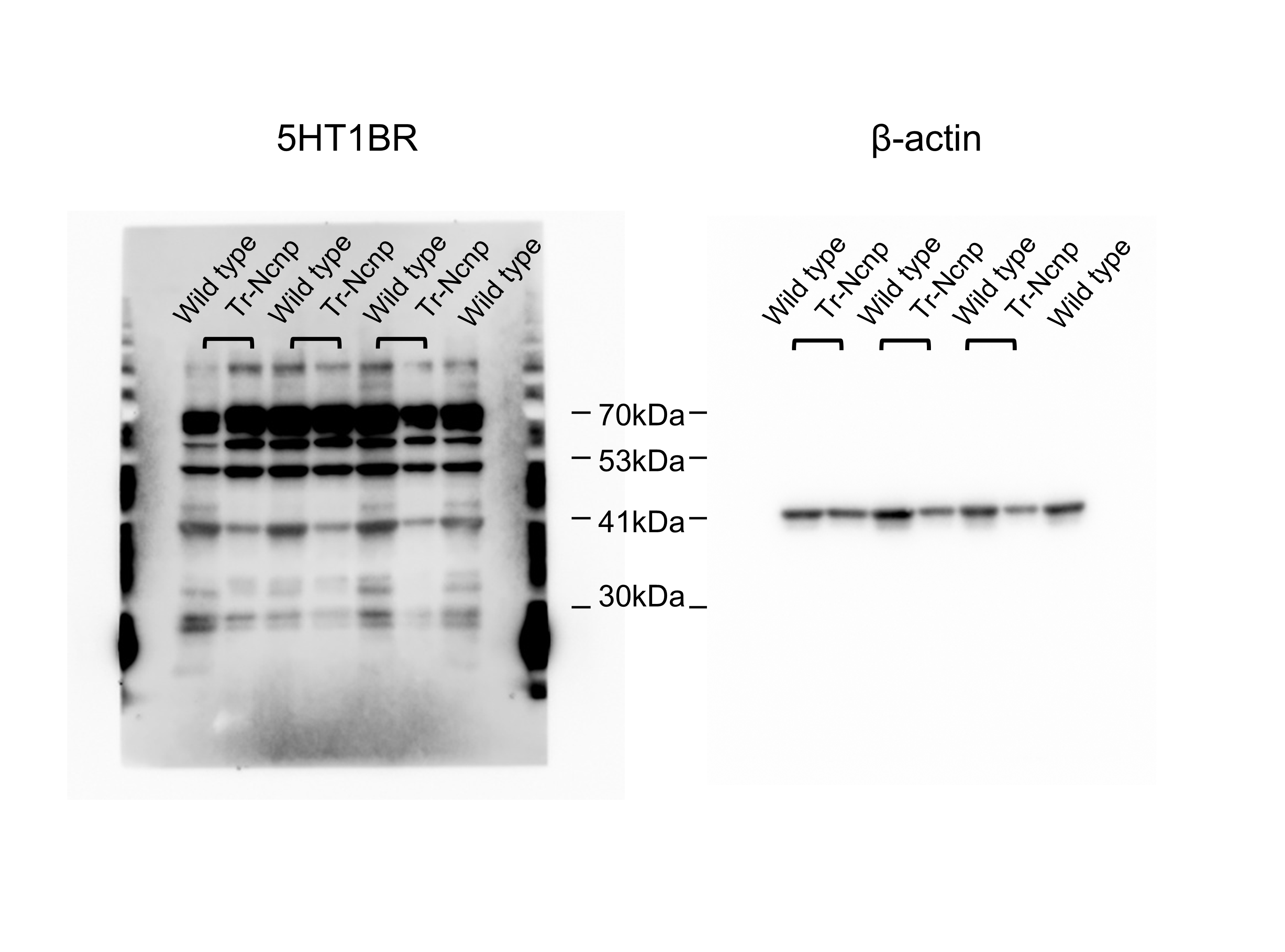

Supplement: S2 Fig — (TIF) [file pone.0345946.s002.tif]
